# The Influence of SARS-CoV-2 Vaccination on the Mortality and Outcomes of Patients with Both Myocardial Infarction and COVID-19

**DOI:** 10.3390/vaccines12090983

**Published:** 2024-08-29

**Authors:** Eugeniusz Hrycek, Anna Walawska-Hrycek, Krzysztof Milewski, Przemysław Nowakowski, Piotr Buszman, Aleksander Żurakowski

**Affiliations:** 1 American Heart of Poland, Topolowa 16, 32-500 Chrzanów, Poland; 2 Department of Cardiology, Faculty of Medical Sciences, Andrzej Frycz Modrzewski Kraków University, 30-705 Kraków, Poland; 3 Department of Neurology, Faculty of Medical Sciences in Katowice, Medical University of Silesia, 40-752 Katowice, Poland; 4 American Heart of Poland, ul. Armii Krajowej 101, 43-316 Bielsko-Biała, Poland; 5 Faculty of Medical Sciences, University of Technology, Rolna 43, 40-555 Katowice, Poland

**Keywords:** COVID-19, vaccination, myocardial infarction, respiratory failure

## Abstract

*Background:* This multi-site retrospective analysis with a control group was devised to evaluate the impact of prophylactic SARS-CoV-2 vaccination the on outcomes of myocardial infarction (MI) patients with confirmed COVID-19. *Methods:* An overall of 129 subjects who had been diagnosed with COVID-19 and MI were included in the analysis and were divided into the study group (44 vaccinated patients) and the control group (85 non-vaccinated comparable patients). The primary outcome measure was defined as the time until in-hospital death, while the secondary outcome measure was defined as the time until death outside the hospital setting. *Results:* According to in-hospital mortality analysis, 1 (2.27%) subject died in the study group, whereas a total of 19 (22.4%) subjects died among the controls (OR = 0.08; CI: 0.001–0.553; *p* = 0.023). The impact of vaccination on the in-hospital outcomes of patients treated for COVID-19 and MI was further confirmed using Cox regression analysis (HR: 0.1 CI: 0.01–0.77; *p* = 0.026). The observed difference was the absence of respiratory failure requiring mechanical ventilation in the study group, whereas it was observed in 14 (16.47%) patients in the control group. During out-of-hospital observation, there were no observed differences in mortality (OR: 1.56; 95% CI: 0.21–11.52; *p* = 0.66). *Conclusions:* The complete prophylactic SARS-CoV-2 vaccination course demonstrates a protective role in patients undergoing treatment for MI with confirmed COVID-19 during in-hospital observation.

## 1. Introduction

Coronavirus disease 2019 (COVID-19) is a well-established cause of lethal pneumonia. It was initially characterized in 2019 in Wuhan, China. The disease is triggered by severe acute respiratory syndrome coronavirus 2 (SARS-CoV-2) and progresses through three distinct stages: initial infection, pulmonary involvement (Stage IIa without hypoxia or Stage IIb with hypoxia), and systemic hyperinflammation, also known as a cytokine storm.

SARS-CoV-2 can affect multiple organs, including the cardiovascular system, leading to indirect injuries such as an increased risk of thromboembolic events (e.g., myocardial infarction, pulmonary embolism, peripheral artery embolism, and disseminated intravascular coagulation) and direct injuries such as myocarditis.

The pandemic impacted healthcare access, resulting in a global reduction in acute coronary syndrome (ACS) admissions [1,2,3,4,5,6,7], extended the time to contact with health services, and delayed the initiation of cardiovascular interventions [8,9,10,11,12,13,14,15]. As a consequence, an increase in heart muscle injury, as confirmed by magnetic resonance imaging [16], and an escalation in the rate of mechanical complications related to myocardial infarction were observed.

Some studies have shown that severe acute respiratory syndrome coronavirus 2 alone can be treated as a predisposing factor of MI [17]. Finally, it has been demonstrated, including through our own studies, that COVID-19 increases the mortality rate in patients hospitalized simultaneously for MI [18,19,20,21,22,23,24,25].

Since the onset of the pandemic, vaccination has emerged as a common method to mitigate the risk of death in COVID-19 patients. The protective effect of vaccination was revealed in the following subgroups of patients with underlying diseases: in patients with type 2 diabetes mellitus (managed by general practitioners), patients with chronic obstructive pulmonary disease, hemodialysis patients, patients with immunodeficiency, and renal transplant recipients [26,27,28,29,30].

Taking into account these considerations, a multi-site retrospective analysis with a control group was devised to evaluate the impact of vaccination on outcomes for myocardial infarction (MI) patients undergoing treatment for COVID-19. The research was also conducted to evaluate the safety of vaccines [31,32]. The presented article is a continuation of our previous study, “The Influence of SARS-CoV-2 Infection on Acute Myocardial Infarction Outcomes”, where COVID-19 patients with myocardial infarction (MI) were compared to those with MI alone. This study was conducted to assess the influence of vaccination on the outcomes of patients with myocardial infarction who were simultaneously treated for SARS-CoV-2 infection.

## 2. Materials and Methods

### 2.1. Study Group

A total of 190 COVID-19 diagnosed patients treated for MI were selected from 10,008 patients treated for MI in 11 American Heart of Poland Cardiology Departments which are units that specialize in the interventional treatment of ACS. The recruitment period was contained to the initial three years of the pandemic. The vaccination status was established based on medical records. The full minimal vaccination course was defined as obtaining at least two doses of the mRNA vaccines, Pfizer-BioNTech (Comirnaty) (New York, NY, USA; Mainz, Germany) or Moderna (Spikevax) (Cambridge, MA, USA), and at least one dose of the viral vector vaccine Johnson & Johnson (Janssen) (New Brunswick, NJ, USA). Hospitalization histories were analyzed retrospectively. The inclusion criteria were defined as follows:

- Confirmed MI, according to the ESC Fourth Universal Definition of Myocardial Infarction [33];
- Confirmed COVID-19 based on a PCR-positive test;
- Verified vaccination status with government-approved vaccines;
- Available after discharge a minimum observation period of 1 month (for patients who were discharged to allow us to create reasonable follow-up);
- Age > 18 years.

The subsequent exclusion criteria were defined as follows:

- Any coexisting disease potentially limiting lifetime during observation (e.g., end-stage organ failure or end-stage cancer);
- Lack of primary clinical data necessary for groups comparison, e.g., glomerular filtration rate (GFR) or ejection fraction (EF).

All the data were verified with medical records. 

A total of 61 participants were not considered for inclusion in the study because of the following reasons: unknown vaccination status (14 patients, 22.9%), end-stage disease (2 patients, 3.3%), lack of minimum observation time (12 participants, 19.7%), and incomplete medical records (23 patients, 37.7%). Additionally, 10 patients were excluded from the study because their clinical characteristics were very different from the study group (to obtain a comparable study and control group). The utilized database was created based on standard care procedures; therefore, ethical approval was omitted.

### 2.2. Study Outcomes

The primary aim of the study was to assess the effect of complete vaccination on in-hospital mortality among patients undergoing treatment for myocardial infarction (MI) and concurrently infected with SARS-CoV-2. The subsequent secondary aims were defined: to assess the impact of vaccination on out-of-hospital mortality among patients undergoing treatment for MI and COVID-19 concurrently and to compare mortality rates before and after the introduction of the population vaccination program. The primary outcome measure was defined as the time until in-hospital death, while the secondary outcome measure was defined as the time until death outside the hospital setting.

### 2.3. Methods

**Figure 1:**
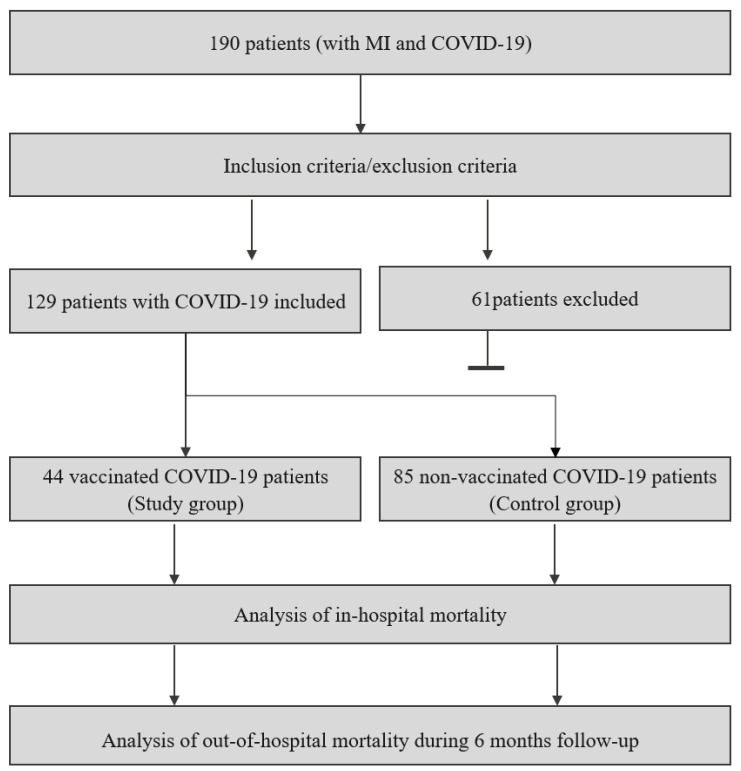
Study design. COVID-19: coronavirus disease 2019, MI: myocardial infarction.

Figure 1 illustrates the study design.

All clinical data were obtained retrospectively from the American Heart of Poland patient database. The study group comprised vaccinated patients hospitalized for both MI and COVID-19, while the controls included non-vaccinated participants admitted for MI and SARS-CoV-2 infection. Then, a subsequent investigation was performed. To assess the primary objective of the study, we compared overall in-hospital mortality between the study group and the control group. This analysis was extended to identify outcome predictors. Finally, out-of-hospital mortality and overall mortality (including in-hospital and out-of-hospital observations) were assessed in the study group compared to the controls.

### 2.4. Statical Analysis and Utilized Software

Percentages were used for presenting categorical variables. Additionally, for continuous variables, means and standard deviations were utilized for parametric data presentation, whereas medians with interquartile ranges were used for nonparametric data. A Shapiro–Wilk test was used to assess data distribution. Student’s t-test was applied for analyzing data with a normal distribution. A Mann–Whitney U test was used for analyzing data with a nonparametric distribution. A Chi-square or Fisher’s exact test was utilized for analyzing differences in proportions. For Fisher’s exact test, the survival differences were illustrated using Kaplan–Meier plots. For the assessment of the relationship between survival time and predictor variables, Cox proportional hazard models were utilized. The study required at least 109 cases to be included for multivariable Cox analysis (based on Peduzzi formula) [34]. The thresholds for the *p* value were set at *p* < 0.05 and *p* < 0.001 for statistically significant results and highly statistically significant results, respectively. 

The PQStat v1.8.4.142 software from PQStat and MedCalc v 22.032 software from MedCalc were utilized for statistical analysis. Artificial intelligence tools such as ChatGPT (versions 3.5 and 4.0) and Grammarly were used only for minor language and grammar corrections. Each sentence was carefully reviewed after correction to ensure it retained the original meaning.

### 2.5. Rationale for Study Design

This article discusses the influence of SARS-CoV-2 vaccination on the outcomes of patients simultaneously being treated for COVID-19 and MI. The presented data provide rare evidence that COVID-19 vaccination impacts the in-hospital mortality of subjects treated for both MI and COVID-19. Based on cited literature (which presents similar observations in other diseases) and the lack of similar observations in patients treated for MI, this issue seems worth further exploring.

## 3. Results

### 3.1. Study Group

Overall, 190 subjects treated for MI also suffered from SARS-CoV-2 infection (18.98 per 1000 subjects with MI, with a frequency rate of 1.9%). A total of 129 (67.9%) subjects were enrolled in the study for analysis. The study group contained 44 patients, including 32 men (72.73%), 12 women (27.27%), 15 patients with STEMI (34.09%), and 29 patients with NSTEMI (65.91%). The control contained 88 non-vaccinated patients with MI and with COVID-19. The study group and the control group were similar in terms of age, gender ratio, MI type, presence of DM, EF, and kidney parameters. The vaccination rate reached 34%. Table 1 presents a detailed comparison of the patient characteristics.

The groups were also comparable in terms of interventional and optimal medical treatment. Only with respect to CABG qualification, multivessel coronary artery disease presence, and staged revascularization were there slight differences noted. It is likely that these differences result from a small numbers effect. Table 2 presents a detailed comparison of the applied treatments.

Table 3 presents a detailed comparison of non-lethal hospital stay events. The only observed difference was the absence of respiratory failure requiring mechanical ventilation in the study group, whereas it was observed in 14 (16.47%) patients in the control group.

### 3.2. Mortality

**Figure 2:**
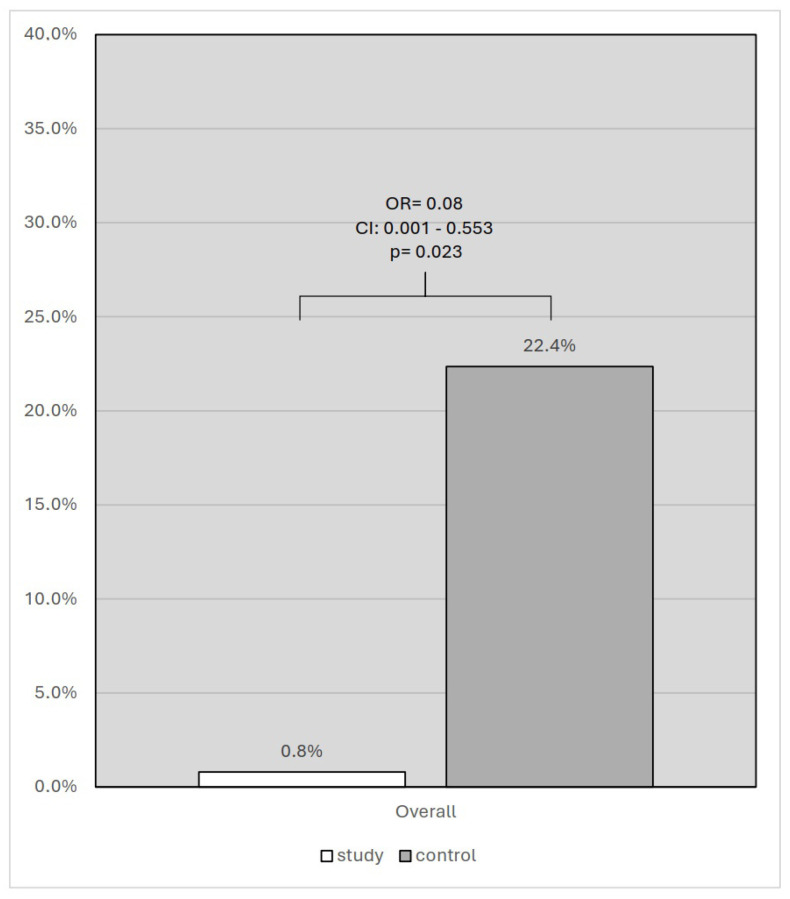
Analysis of in-hospital mortality: study group vs. controls.

In the study group, 1 (2.27%) patient died, whereas 19 (22.4%) patients died in the control group (OR = 0.08; CI: 0.001–0.553; *p* = 0.023; Figure 2).

The impact of vaccination on in-hospital outcomes of patients treated for COVID-19 and MI was further confirmed using Cox regression analysis (Table 4). Among other assessed factors, only the presence of DM reached significance as a predictor of outcome. It is important to note that in the study being discussed, the year of the pandemic (during which patients were affected by COVID-19) did not significantly predict the outcome.

**Figure 3:**
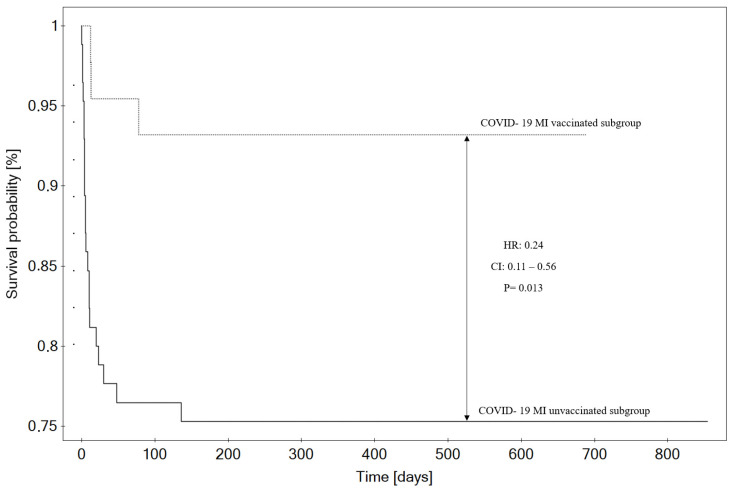
Kaplan–Meier’s analysis of overall mortality (including in-hospital and out-of-hospital data).

During out-of-hospital observation, two (4.88%) patients died in the study group, and two patients (3.03%) died in the control group (OR: 1.56; 95% CI: 0.21–11.52; *p* = 0.66). During the overall observation period (including both in-hospital and out-of-hospital data), 3 (6.82%) patients died in the study group, whereas 21 (24.71%) patients died in the control group (HR: 0.24; 95% CI: 0.11–0.56; *p* = 0.013; see Figure 3).

An additional evaluation was conducted to assess the lethal outcome rates before and after the implementation of the population vaccination program. The subgroup of patients before the mass vaccination consisted of 28 individuals, among whom 7 patients died, resulting in a mortality rate of 25%. In the period following the introduction of vaccinations, the subgroup comprised 101 patients, with 13 deaths reported, yielding a mortality rate of 13%. No statistically significant difference was observed between the subgroups of patients mentioned above (OR: 2.24; CI: 0.67–6.98; *p* = 0.11). The same comparison was conducted after exclusion of 44 vaccinated patients. In the period following the introduction of vaccinations, the subgroup of unvaccinated comprised 57 patients, with 12 deaths reported, yielding a mortality rate of 21%; this result also did not reach statistical significance (OR: 1.00; CI: 0.02–39.00; *p* = 0.39). It is important to interpret these results cautiously due to the relatively small sample sizes in groups.

## 4. Discussion

Based on the presented data, the following observations were formulated. In our study (among overall MI patients), the frequency of COVID-19 was higher than in the cited studies (1.9%). On the other hand, the epidemiological studies revealed that up to 1% of subjects treated for STEMI can suffer from SARS-CoV-2 infection [35]. This observation could be a result of the repeated PCR testing. In the presented study, the fatal outcome rate in the study group (vaccinated MI patients with COVID-19) was noticeably lower, comparable with the mortality of MI patients without COVID-19. In the case of MI patients, the in-hospital fatal outcome rates reached 7% and 4.9% in STEMI and NSTEMI groups, respectively [36]. The mortality in the control group was similar to the results obtained by other authors assessing the influence of SARS-CoV-2 infection on the in-hospital outcomes of MI patients. In patients treated for both MI and SARS-CoV-2 infection, a kind of accumulation of death risk was observed. The fatal outcome rate in subjects treated for both SARS-CoV-2 infection and MI seems to be higher in both non-hospitalized and hospitalized patients treated for COVID-19, ranging from 10% to even 76.6% [18,19,20,21,22,23,24,25].

It is worth mentioning that the COVID-19 fatal outcome rate in the overall population is approximately 3.4%. The protective effect of vaccination has been confirmed worldwide in population and international studies assessing the mortality of COVID-19 [37,38]. Based on the data from these population studies, the mortality difference (delta) between vaccinated and unvaccinated patients ranged from 3.2% to 12.7% [38,39]. The mortality rate among hospitalized vaccinated patients was 5.1%, whereas for unvaccinated hospitalized patients, it was 8.3% [39]. Unvaccinated patients are 2.46 times more likely to die from COVID-19 [40]. The protective role of vaccination was previously confirmed in COVID-19 patients suffering for other diseases. The most significant benefits were observed in the subgroups of severely obese hospitalized patients (OR: 0.52) [39]. Similar observations were conducted in other COVID-19 subgroups, including organ transplant recipients during the 90-day follow-up (25% vs. 7.3%), hip fractured patients with COVID-19 (10% vs. 31.2% and 25% vs. 43.75% for 30 days and 90 days post-procedural observation time respectively), intubated patients with COVID-19 (61.5% vs. 68.2%), overall hospitalized patients with COVID-19 (12.5% vs. 31.45%), and intensive care unit patients (56.8% vs. 57.4%) [39,41,42,43,44,45,46,47].

Moreover, additional regulations characterizing the influence of vaccination on the course of COVID-19 have been noted. It has been demonstrated on a population level that the raise of vaccination correlates with decrease in lethal outcomes rates [48]. It has also been revealed that COVID-19 mortality depends not only on the destination vaccination rate but also on vaccination efficiency, which is contingent upon healthcare system organization [49]. Furthermore, even a single dose of vaccine significantly contributes to the reduction in COVID-19-related mortality [50,51]. On a population level across countries, vaccination coverage has been associated with lower all-cause mortality rates. Among the studies cited, only Sangam et al. did not notify a reduction in fatal outcome rates in patients hospitalized for COVID-19 [52]. It is essential to emphasize that comparing the results obtained in the aforementioned studies may be challenging due to the varied statistical methods applied in each study [35,36].

The results obtained were consistent with those of other studies, suggesting that vaccination appeared to be a predictor of favorable in-hospital outcomes. However, the observed reduction in mortality (delta) and odds ratio (OR) were among the highest observed when compared both to the general population of COVID-19 patients and to the selected populations of COVID-19 patients mentioned above (the delta in the presented study was 21.6%, while the delta in the general population of hospitalized patients was 3.2%). These results may indicate that myocardial infarction (MI) patients suffering from COVID-19 may particularly benefit from vaccination. It also suggests that the hospital outcomes of MI patients treated for COVID-19 are strongly dependent on COVID-19. Unlike most other studies, the presented study included out-of-hospital follow-up.

It was observed that the protective effect of vaccination in subjects suffering from both MI and SARS-CoV-2 infection appears only during hospitalization and does not extend to the post-hospitalization period. According to the comparison of mortality rates before and after the introduction of the population vaccination program, the obtained results indicate that the vaccination rate of 34% was insufficient to cause a statistically significant reduction in mortality after applying the population vaccination program in the group of patients treated simultaneously for MI and COVID-19. It is also necessary to mention that based on meta-analysis, it has been demonstrated that COVID-19 vaccination can cause a transient, short-term, slight increase in the risk of cardiovascular events [53]. Conversely, based on the obtained results, a significant short-term protective effect of vaccination during the acute phase of MI (secondary prevention) was observed.

## 5. Study Limitations

Sixty-one subjects were not included in the study, primarily due to a lack of basic information, making it difficult to compare their outcomes. The results might have been biased due to the relatively small size of the study group. The study did not attain sufficient statistical power to include the Grace score in the Cox model analysis. The multiple comparisons between the study and control groups were not conducted because most of the obtained data did not follow a normal distribution. A group of patients who had completed the minimum recommended course of vaccination with government-approved vaccines was identified based on medical records. However, determining the proportions of applied specific vaccines was impossible due to the lack of appropriate data.

## 6. Conclusions

During hospitalization, the complete vaccination course demonstrates a protective role in patients undergoing treatment for MI and COVID-19. The increased occurrence of respiratory failure accounts for the higher mortality rate observed among COVID-19 patients treated for MI who did not complete the entire vaccination course.

## Figures and Tables

**Table 1:** Baseline data.

	Study Group (n = 44)	Control Group (n = 85)	*p*
Male, n (%)	32 (72.73%)	59 (69.41%)	0.70
STEMI, n (%)	15 (34.09%)	43 (50.59%)	0.08
Age, years	67 (60–75)	71 (61–76.5)	0.36
EF, %	45 (35–50)	50 (40–55)	0.053
GFR, mL/min	78.8 (56–95.77)	74.39 (47.77–97.92)	0.67
Diabetes, n (%)	13 (29.55%)	23 (27.06%)	0.77
Hypertension, n (%)	31 (70.45%)	60 (70.59%)	0.99
Hyperlipidemia, n (%)	27 (61.36%)	36 (42.35%)	0.05
Smoking, n (%)	8 (18.18%)	14 (16.47%)	0.81
Peripheral arterial disease, n (%)	2 (4.55%)	1 (1.18%)	0.23
Obesity, n (%)	3 (6.82%)	12 (14.12%)	0.22
Previous stroke, n (%)	3 (6.82%)	6 (7.06%)	0.96
Atrial fibrillation, n (%)	6 (13.64%)	14 (16.47%)	0.68
Myocardial infarction, n (%)	11 (25%)	13 (15.29%)	0.18
Previous PTCA, n (%)	8 (18.18%)	11 (12.94%)	0.43
Previous CABG, n (%)	5 (11.36%)	1 (1.18%)	0.01
Time from vaccination to COVID-19, days	257 (201.5–294.5)		

COVID-19: coronavirus disease 2019; EF: ejection fraction; GFR: glomerular filtration rate; STEMI: ST-elevation myocardial infarction.

**Table 2:** Treatment and culprit lesions.

	Study Group (n = 44)	Control Group (n = 85)	*p*
Coronarography, n (%)	43 (97.73%)	82 (96.47%)	0.70
Optimal medical therapy, n (%)	1 (2.27%)	3 (3.53%)	0.70
Coronary artery bypass grafting qualified, n (%)	6 (13.64%)	3 (3.53%)	0.03
Percutaneous transluminal coronary angioplasty, n (%)	32 (72.73%)	73 (85.88%)	0.07
Reached TIMI 3, n (%)	30 (93.75%)	62 (84.93%)	0.15
Multivessel coronary artery disease, n (%)	16 (36.36%)	15 (17.65%)	0.02
Left main coronary artery, n (%)	1 (2.27%)	2 (2.35%)	0.73
Left anterior descending artery, n (%)	13 (29.55%)	25 (29.41%)	0.99
Diagonal branches, n (%)	3 (6.82%)	2 (2.35%)	0.21
Circumflex artery, n (%)	4 (9.09%)	7 (8.24%)	0.87
Obtuse marginal artery, n (%)	2 (4.55%)	3 (3.53%)	0.78
Right coronary artery, n (%)	7 (15.91%)	21 (24.71%)	0.25
Posterior descending artery, n (%)	0 (0%)	0 (0%)	
Intermediate artery, n (%)	0 (0%)	1 (1.18%)	0.47
Bridge, n (%)	1 (2.27%)	1 (1.18%)	0.64
Stent length, mm	32.5 (23.25–57.5)	32 (21.5–48)	0.45
Staged revascularization *, n (%)	4 (9.09%)	1 (1.18%)	0.03

TIMI3: thrombolysis in myocardial infarction, * multiple interventions conducted during hospitalization.

**Table 3:** In-hospital non-lethal events.

	Study Group (n = 44)	Control Group (n = 85)	*p*
Cardiogenic shock, n (%)	3 (6.82%)	11 (12.94%)	0.32
Pulmonary edema, n (%)	5 (11.36%)	5 (5.88%)	0.30 *
Respiratory failure, n (%)	0 (0%)	14 (16.47%)	0.02 *
Contrast-induced nephropathy, n (%)	3 (6.82%)	11 (12.94%)	0.29
Stroke, n (%)	0 (0%)	0 (0%)	
Bleeding requiring transfusion, n (%)	0 (0%)	4 (4.71%)	0.30 *
Hospitalization time (days)	10.67 ± 8.78	9.13 ± 5.62	0.29

* by Fisher’s exact test.

**Table 4:** Cox proportional hazards model for identifying predictors of all-cause mortality.

	Univariable Cox Regression				Multivariable Cox Regression			
Parameter	HR	−95% CI	+95% CI	*p*	HR	−95% CI	+95% CI	*p*
Vaccination	0.11	0.01	0.86	0.036	0.10	0.01	0.77	0.026
Sex	1.33	0.48	3.71	0.584				
Diabetes	2.39	0.99	5.78	0.052	2.76	1.14	6.66	0.024
GFR, mL/min	0.98	0.97	1.00	0.101				
EF, %	0.96	0.91	1.00	0.063				
Age, years	1.02	0.99	1.06	0.206				
MI type (STEMI vs. NSTEMI)	0.76	0.30	1.09	0.56				
Year of pandemic (1–3)	0.58	0.28	1.18	0.12				

EF: ejection fraction; GFR: glomerular filtration rate; MI: myocardial infarction; NSTEMI: non-ST-elevation myocardial infarction; STEMI: ST-elevation myocardial infarction.

## Data Availability

Derived data supporting the findings of this study are available from the corresponding author on request.
